# Healthcare professionals' perception and satisfaction with mental health tele-medicine during the COVID-19 outbreak: A real-world experience in telepsychiatry

**DOI:** 10.3389/fpsyt.2022.981346

**Published:** 2022-11-04

**Authors:** Carlos Roncero, Diego Remon-Gallo, Nerea Casado-Espada, Lourdes Aguilar, Sinta Gamonal-Limcaoco, María Teresa Gallego, Berta Bote, Angel Luis Montejo, Barbara Buch-Vicent

**Affiliations:** ^1^Psychiatric Service, University of Salamanca Health Care Complex, Salamanca, Spain; ^2^Salamanca Institute of Biomedicine, University of Salamanca, Salamanca, Spain; ^3^Psychiatric Unit, School of Medicine, University of Salamanca, Salamanca, Spain; ^4^School of Nursing, University of Salamanca, Salamanca, Spain; ^5^Faculty of Psychology, University of Salamanca, Salamanca, Spain

**Keywords:** tele-medicine, healthcare professional's perception, COVID-19, pandemic, telepsychiatry, e-health

## Abstract

**Background:**

The use of telemedicine is increasingly being implemented, showing numerous benefits over other methods. A good example of this is the use of telemedicine following the breakdown caused by the COVID-19 pandemic. Previous experiences with telemedicine (TM) have not been significantly explored in relation to the professionals' own perspectives.

**Objective:**

Identify and explore the perceptions and interests of mental health professionals who have performed TM during the period of pandemia.

**Methods:**

A questionnaire on mental health professionals' perceptions of and satisfaction of TM, the Font Roja Work Satisfaction Questionnaire, was adapted and used. Data collected included 112 Psychiatric Service professionals who conducted TM in March 2020, after the country had been under lockdown for 10 weeks. Over 12.000 medical consultations were carried out by the phone, showing an overwhelming response to this method.

**Results:**

High levels of satisfaction were recorded amongst professionals. TM would function as a complement to the traditional system of face-to-face visits (n-112, f-109, 96.5%). Only 9.7% (f-11) believed that digital or virtual interventions would completely replace face-to-face visits. 60.8% did not consider this monotonous work. The older the health workers were, the more satisfied they felt during their follow-up telephone consultation. The greater the previous experience, the more satisfaction was shown. There were gender differences: female mental health workers reported a greater level of comfort.

**Conclusion:**

TM can be implemented with less effort, but it requires time, methods, and resources to be managed. Satisfaction among professionals is high, especially among those with more clinical experience. Patient satisfaction must be contrasted against this.

## Introduction

The COVID-19 pandemic is the most critical public health issue this society has faced in the last 50 years. By October 27, 2021, more than 244 million people have been infected worldwide (5006675 in Spain), with 4968445 deaths (1, 2). This eventful issue forced mental health services to quickly modify their usual procedures to face the new situation (3, 4). The National Health System in Spain is made up of a set of health services dependent on public authorities. It is free and universal: consultations, access to emergencies and medicines are free. For this reason, the majority of Spaniards turn to the public health system on a regular basis. The method usually used is face-to-face attendance. Telemedicine (TM) has become an essential tool to guarantee mental health in this new situation (4, 5). However, it must be carefully analyzed to be considered effective in the future because as experiences in this field are remain limited (6, 7), even though they have increased in recent years. Little is known about the subjective perception, satisfaction, and interest in TM of mental health professionals who have conducted it both in a the pre-pandemic context (8) and during the pandemic outbreak (4, 5). Knowing professionals' perspectives is valuable since they diagnose and treat diseases based on the resources, they have access to (9–11). Based on these facts, TM's use should be planned and implemented for future scenarios or clinical development for the post-COVID-19 stage.

There are increasingly more studies based on the assistance provided during the pandemic COVID-19 (12–14) however, this study highlights the point of view of the professionals who have performed these services. From this perspective, gender differences among mental health professionals still remain under-researched and under-reported, despite the fact that most of the task force in mental health are women (2).

This study describes how mental health professionals of the Psychiatry Department at the University of Salamanca Health Care Complex (USHCC) adapted and perceived TM after switching from the traditional face-to-face model to the new widely-available TM system on March 16 2020, after the lockdown that lasted 10 weeks (15). We also focus on socio-demographic and gender differences, factors that must be analized (16). This point of view is interesting because it helps to analyze the perceived self-efficacy of the workers, and the difficulties they encounter in their work, and contributes to improving the quality of the services provided.

Based on previous information, the main hypotheses are:

- Telemedicine acceptance and satisfaction will increase among professionals exposed to this modality (way of working).

- Professionals carried out tele-matic consultations (telemedicine) will be more willing to use it as part of their future practice.

- Professionals' perception of patients' satisfaction will be more significant in patients attended by more satisfied and experienced professionals.

- Age or gender differences exist in professional satisfaction when applying telemedicine.

## Materials and methods

Data were obtained from a sample of mental health professionals of the Psychiatry Service (PS) of the USHCC, including 45 different medical services and more than 900 beds. The PS includes most Salamanca Mental Health Network resources, including part of the drug dependence care network, community outpatient services, inpatient units and community flats. It also includes psychiatric emergency services and on-call doctors at the University Hospital (UH), plus a transversal program for suicide prevention and attention for refractory mental disorders.

**Figure 1:**
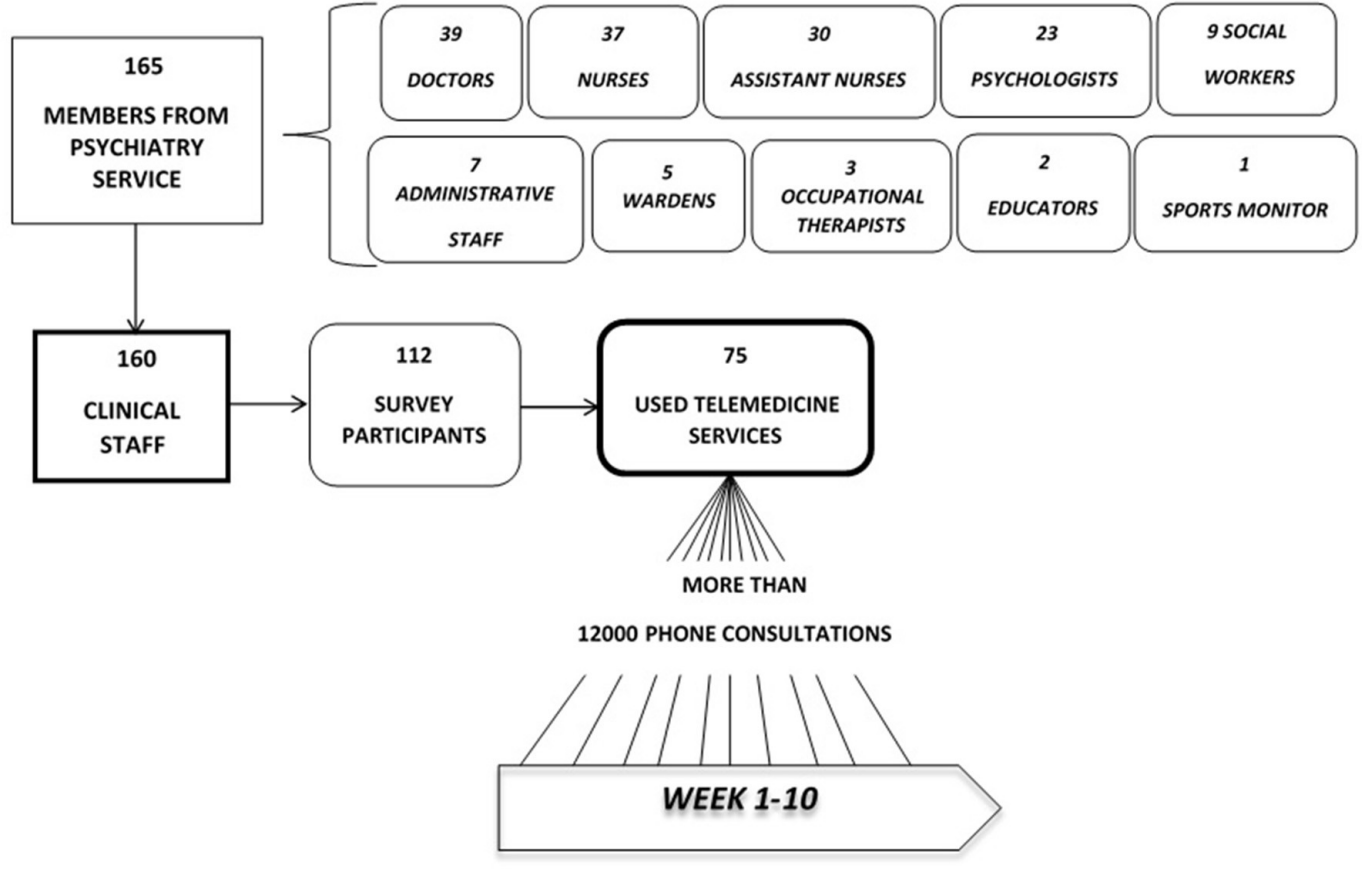
A summary of the data collection.

One hundred and twelve participants participated in the study, 75 of them provided TM services while confined. During the first 10 weeks of the COVID-19 outbreak, over 12,000 medical consultations were conducted by phone. A summary of the data collected can be found in Figure 1.

Each professional was able to participate in the study; however, those professionals who did not apply TM only answered 18 questions from the questionnaire.

An observational descriptive, cross-sectional study was carried out to understand TM's expectations and satisfaction by PS members during the COVID-19 pandemic confinement. The analysis was performed in a single phase using a questionnaire developed and carried out by the authors.

A questionnaire on mental health professionals' perceptions and satisfaction of TM, the Font Roja Work Satisfaction Questionnaire, was adapted and used. The authors designed an adapted questionnaire based on the literature's references, including 49 closed-ended questions. This questionnaire includes some questions, grouped into nine factors, which exploring different areas involved in job satisfaction: (1) work monotony; (2) job satisfaction; (3) stress/stress from work; (4) competition; (5) labor pressure; (6) promotional capacity; (7) relationship with superiors; (8) relationship with colleagues; (9) extrinsic characteristics of work (17). Four additional items were included: 2 to assess the work environment (18) and 2 to measure the professional's perception of the patient's satisfaction. The user satisfaction was measured on a scale of 1 (not at all satisfied) to 10 (maximum satisfaction). An additional open question was added at the end of the questionnaire to include qualitative information and personal feedback.

The online questionnaire was sent by email to all the staff members on May 15, 2020, and was available online until May 25, 2020. There were no exclusion criteria; every member from the PS received the e-mail. The procedures followed ethical standards and the Organic Law 3/2018, of the 5th of December, on the Personal Data Protection guaranteeing digital rights. The questionnaire was confidential, and subjects accepted by informed consent with the possibility to withdraw voluntarily at any time.

For quantitative data, normality tests were performed through either the Kolmogorov- Smirnov or Shapiro-Wilk Test. The described data was measured with the dispersion measures of mean and standard deviation for normal samples and the Median and Interquartile Range (IQR) for models that did not comply with the normality criteria.

Qualitative variables were explained with tables of good percentage or frequencies, which corresponded to be more appropriate. For parametric variables, an analysis was performed through either the Student's *T*-test for independent sample differences or the ANOVA test, when more than two groups existed. Mann-Whitney's *U*-test was used for non-parametric variables for two independent groups or Kruskal-Wallis when there were more than two separate groups. Qualitative variables were correlated by Fisher or Chi-Square tests, depending on which was appropriate in each case. The results were considered statistically significant for all analyses when the *p*-value was < 0.05 (significance level α = 0.05). SPSS software, v.26.0, was used for Mac.

## Results

### Sample description

The sample involved 112 participants, representing 70% of the PS total members (*n* = 160). 82.1% of the sample was working during COVID-19, and 66.4% carried out TM. In this sub-sample, as data to mention, 13 subjects (11.6%) lived with people who had COVID-19 while working remotely. Furthermore, 84.69% of all those who carried out TM, had never done TM or did without previous training.

On another front, concerning other professionals' characteristics, it should be said that most of the subjects who performed telemedicine were Doctors (32.1%) and 20.5% of the sample had management responsibility. The professionals were homogeneously distributed by different age groups and years of experience; however, gender differences were not found in the distribution of the sample (Table 1).

**Table 1:** Sociodemographic and professional profile among respondents.

	**Total *n* = 112%**	**Women *n* = 85%**	**Men *n* = 27%**
Age			
< 30 31-40 41-50 51-60 >60 Lost data	12 (10.7) 23 (20.5) 17 (15.7) 29 (25.9) 23 (20.5) 8 (7.1)	8 (10.1) 22 (27.8) 11 (13.9) 20 (25.3) 18 (22.8) 6 (7.05)	4 (16.0) 1 (4.0) 6 (24.0) 9 (36.0) 5 (20.0) 2 (7.4)
Professional category			
Doctors Psychology Nursing Social worker& educators Nursing assistant Occupational therapist Administrative staff	36 (32.1) 24 (21.4) 21 (18.8) 11 (9.8) 10 (8.9) 3 (2.7) 7 (6.3)	24 (28.2) 17 (20.0) 19 (22.4) 10 (11.8) 9 (10.6) 2 (2.4) 4 (4.7)	12(44.4) 7 (25.9) 2 (7.4) 1 (3.7) 1 (3.7) 1 (3.7) 3 (11.1)
Working experience (number of years)			
<8 9-16 17-24 25-32 >33	26 (31.8) 26 (25) 11 (11) 17 (17) 21 (21)	29 (34.5) 21 (25) 5 (6) 12 (14.3) 17 (20.2)	8 (29.6) 5 (18.5) 6 (22.2) 4 (14.8) 4 (14.8)
Family situation during confinement			
Living alone Reconciling family and work-life Lost data	15 (13.4) 95 (84.8) 2 (1.8)	12 (14.3) 72 (85.7) 1 (1.2)	3 (11.5) 23 (88.5) 1 (3.7)

Regarding the TM method used, there were no prior resources or data protection systems to facilitate videoconferencing be realized, so the telephone was the most popular. Medical consultation by phone is considered as an immediate and direct method of telematic assistance, which allows the user to contact the health professional from anywhere and in any circumstance. Most subjects tracked digital follow-up used this method, followed by chat/email (40%) and video calling (4/75, 9.3%). Most of health professionals who used these methods had direct access to the hospital health platforms, and could carry out the health service with computer support to consult and update the medical history of the patients.

It could be said the amount of TM accomplished was significant as 32 % of the sample made more than 40 connections per week.

### Professional's subjective perceptions about telemedicine and the future

This new TM service was considered an innovative practice for most participants. Most full sample participants (85%) pointed out that TM would increase its use in the coming years and found it very useful. Meanwhile, 60% of the sample thought that TM was not yet well-defined and 4.4% of the sample felt that this option would not be implemented and did not consider it worthwhile. Despite the fact that only eight subjects (7.1%) stated that TM would not cause any side effects, most professionals thought TM would complement the traditional face-to-face visits model (97.3%). Furthermore, some gender differences were found (Table 2).

**Table 2:** Professional's perceptions about TeleMedicine.

	**Total *n* = 112%**	**Women *n* = 85%**	**Men *n* = 27%**
Time off work during confinement			
Yes No Lost data	19 (17) 92 (82.1) 1 (0.9)	16 (19) 68 (81) 1 (1.2)	3 (11.1) 24 (82.9) 0
Cohabitation with COVID-19 infected people during confinement			
Yes No Not applicable	13 (11.6) 96 (85.7) 3 (2.7)	10 (11.8) 72 (84.7) 3 (3.5)	3 (11.1) 24 (88.9) 0
Management responsibility			
Yes No	23 (20.5) 89 (79.5)	14 (16.5) 71 (83.5)	9 (33.3) 18 (66.7)
Telemedicine previous experience			
Yes No	50 (44.6) 62 (55.4)	35 (41.2) 50 (58.8)	15(55.6) 12(44.4)
Future perspectives about telemedicine implementation			
Increased use Decreased use No variation	96 (85.7) 5 (4.5) 11 (9.8)	74 (87.0) 2 (2.4) 9 (10.6)	22 (81.5) 3 (11.1) 2 (7.4)
Appropriate resources are available for Telemedicine			
1. Totally disagree 2. Partly disagree 3. Neither agree nor disagree 4. Partly agree 5. Totally agree Lost data	21 (18.8) 39 (34.8) 45 (40.2) 4 (3.6) 2 (1.8) 1 (0.9)	12 (14.1) 29 (34.1) 39 (45.9) 4 (4.7) 1 (1.2) 0	9 (34.6) 10(38.5) 6 (23.1) 0 (0) 1 (3.8) 1 (3.7)
Telemedicine is regulated appropriately			
1. Totally disagree 2. Partly disagree 4. Partly agree 5. Totally agree Lost data	24 (21.4) 45 (40.2) 37 (33.0) 5 (4.5) 1 (0.9)	15 (17.9) 33 (39.3) 32 (38.1) 4 (4.8) 1 (1.2)	9 (33.3) 12(44.4) 5 (18.5) 1 (3.7) 0
Telemedicine could have secondary effects			
1. Totally disagree 2. Partly disagree 3. Neither agree nor disagree 4. Partly agree 5. Totally agree Lost data	8 (7.1) 28 (25.0) 46 (41.1) 17 (15.2) 12 (10.7) 1 (0.9)	5 (6.0) 25 (29.8) 34 (40.5) 11 (13.1) 9 (10.7) 1 (1.2)	3 (11.1) 3(11.1) 12(44.4) 6 (22.2) 3 (11.1) 0
Telemedicine could replace on-site visits			
Yes No Lost data	11 (9.8) 99 (88.4) 2 (1.8)	7 (8.4) 76 (91.6) 2 (2.4)	4 (14.8) 23(85.2) 0
Telemedicine could supplement on-site visits Yes No	109 (97.3) 3 (2.7)	82 (96.5) 3 (3.5)	0 (0) 27 (100) 0

### Job satisfaction perceived during COVID-19

More than half of the respondents (60.8%) did not consider this experience as monotonous work, and this result was significantly related to having prior knowledge of TM (*P* = 0.02).

Concerning professionals' feelings about this practice, in our study, a majority of participants (71.6%) perceived some responsibility when making decisions and 14.9% of the subjects felt very tired at the end of the day, while 50% reported the opposite feeling. Furthermore, 16.3% of the sample had mood disturbance, in comparison to half of the respondents that did not experience any mood alteration (50%) related to the change from face-to-face to TM. Secondly, while a small number of participants (6.8%) indicated an over-strain, 35.5% of participants indicated they could disconnect at the end of the day. In our study, the failure to disconnect at the end of the day has been related to the confinement situation (living alone vs. merging social and family life 90.4%) and a significant relationship between the two variables was found (*p* = 0.041).

Almost all subjects (83.2%) considered themselves independent to do their work, 75.6% were satisfied with their work, 50% felt their work was well-recognized and 68.9% perceived that they had had the opportunity to learn new aspects. The Work Satisfaction Questionnaire category “I have gained recognition for my work” also showed a statistically significant correlation depending on whether the subjects reconciled work and family life or lived alone (*P* = 0.02). Among all the participants, only one perceived that thet could not do their job (1.4%), while 85.1% felt qualified.

Under other matters, 47.3% considered having a salary suitable for the work done and 67.5% an acceptable position. Competitiveness did not appear to be a problem in these circumstances, as only three subjects reported stress or tension because of that fact. Some professionals (39.2%) perceived that they received enough time to organize their work, while 23% lacked time. More than half of the staff (62.1%) knew what was expected of them at work, while the 16.2% had some doubts about their task.

There is a relationship between the category: “how problems of other colleagues can affect you” and the situation of coexistence during the confinement (living alone vs. merging social and family life) *P* = 0.008. It should be noted that 30 subjects of the total sample (*n* = 160) were infected by COVID, although sick leaves did not appear to correlate with any other items (categories).

Relating the extrinsic characteristics of the work, the same number of subjects (36.5%) seemed to have the right resources to perform their work which is statistically related to a better adaptation of the work (*P* < 0.001), better working environment (*P* = 0.002) and the physical structure of work performed at home (*P* = 0.02). Most respondents (67.6%) did not consider that the work's physical structure at home interfered with their ability to carry it out.

Some gender differences were detected in satisfaction showing that females were more prone to TM and more satisfied and adapted (Table 3).

**Table 3:** The satisfaction of the professionals performing telemedicine and differences according to gender.

**Work satisfaction questionnaire of font roja**				
	**Total [Mean (SD)]**	**Men (M)**	**Women (M)**	***P***
“My work does not change. I find it monotonous”.	2.24 (1.01)	2.21	2.25	.76
“I have been responsible for making decisions.”	3.95 (0.91)	3.90	3.96	.71
“I have felt exhausted at the end of the working day.”	3.42 (0.92)	3.50	3.39	.88
“I have not disconnected from work at the end of the working day.”	3.12 (1.25)	3.02	3.40	.18
**“I have had to do my best in my daily work.”**	**3.97 (0.92)**	**3.55**	**4.13**	**.03**
“My work has changed my mood.”	2.39 (1.10)	2.45	2.37	.90
**“I have been satisfied with my work.”**	**3.95 (0.95)**	**3.50**	**4.11**	**.04**
“I have been independent to organize my work.”	4.12 (1.01)	3.95	4.19	.35
“I have had the chance to learn new things.”	3.85 (1.14)	3.50	3.98	.24
“I am interested in the work I have carried out.”	4.23 (0.95)	3.95	4.34	.12
“I have the feeling that the work I have carried out it is no worth.”	1.46 (0.78)	1.60	1.41	.16
**“I have obtained professional recognition for my work.”**	**3.39 (1.12)**	**2.65**	**3.67**	**.002**
“The relationship with my superior has been cordial during this period”	4.43 (0.98)	4.05	4.57	.19
**“The relationship with my co-workers has been cordial during this period”**	**4.53 (0.78)**	**4.20**	**4.65**	**.02**
**“I have the proper salary concerning what I have been doing.”**	**3.14 (1.30)**	**2.05**	**3.54**	**.00**
**“I think I occupy the position I deserve.”**	**3.92 (1.02)**	**3.40**	**4.11**	**.02**
“I think I have the opportunity to progress.”	3.16 (1.11)	3.40	3.07	.19
“I have not had enough time to organize my work.”	2.71 (1.12)	3.15	2.55	.056
“I knew what was expected from my work.”	3.65 (0.97)	3.55	3.69	.52
**“I think my work has been disproportionate.”**	**2.66 (0.96)**	**3.25**	**2.43**	**.006**
“My co-workers' problems have affected me.”	3.16 (1.31)	3.05	3.20	.64
“I often feel I may not be able to carry out my work.”	1.68 (0.76)	1.50	1.74	.16
**“I have not had enough resources to do my work.”**	**3.07 (1.23)**	**3.70**	**2.83**	**.007**
“Competitiveness has been very stressful for me.”	1.72 (0.94)	2.00	1.62	.23
**“My work environment (at home) has prevented me from doing my job satisfactorily.”**	**2.11 (1.31)**	**2.85**	**1.83**	**.01**
“The physical structure of my work environment has interfered with my performance.”	1.96 (1.15)	2.20	1.86	.43
“In general, assess your satisfaction with the telemedicine you have carried out.”	7.41 (1.83)	6.70	7.68	.06
**Patients' satisfaction concerning telemedicine perceived by professionals**
“In general, assess patients' satisfaction concerning the telemedicine carried out.”	7.93 (1.50)	7.60	8.06	.55
**“In general, assess the degree of comfort experimented by the patients concerning the telemedicine.”**	**7.76 (1.45)**	**6.85**	**8.09**	**.009**

M, Mean; SD, Standard deviation.

### Telemedicine general satisfaction

**Figure 2:**
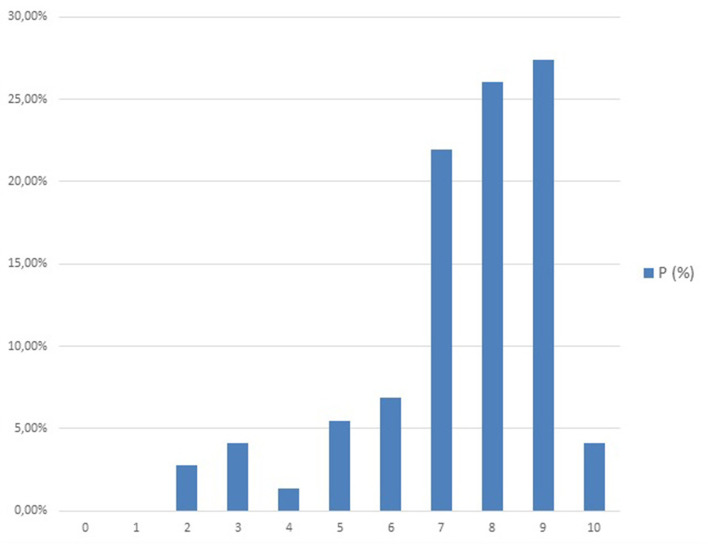
Level of general satisfaction on telemedicine (range 0–10).

Respondents scored a high perceived satisfaction with their work done with TM (Me = 8; IQR = 8 (*n* = 73) being the highest score 9/10 (very satisfied) (Figure 2).

Previous experience with TM seems to be related to more satisfaction in its performance (*P* = 0.029). There were no statistically significant differences between the different professional categories concerning perceived satisfaction when conducting TM. According to our results, satisfaction with telemedicine was also not related to professional responsibility. However, the group with more than 30 years of experience showed the highest satisfaction rates. Linear regression was generated to predict participants' satisfaction with their therapy performed according to their age. The older the health workers were, the more satisfied they felt with their telephone follow-up model. This model would have great goodness of fit index (R-squared-0.94), so it would be appropriate to predict one variable over the other. Moreover, TM's positive opinions for the future were significantly related to the respondents' general satisfaction (*P* = 0.002) and with more social connections, professionals had more satisfaction with the activity performed (*P* = 0.007).

### Professional's perception of patient's satisfaction

The mental health care providers also positively assessed the degree of satisfaction and comfort perceived from the patients receiving TM (Me = 8, IQR = 7 (*n* = 74), indicating that patients were “quite satisfied” with the treatment received. It is remarkable that five participants point out the risk of dehumanization in open questions and that human contact is needed for a good therapeutic relationship. On the other hand, the perceived professional satisfaction of the patients' approval was related to age (*P* = 0.01), according to the previous linear regression model (Me = 8; IQR = 7; average score = 7.76 (DT = 1,451; *n* = 74). This subjective perception of the patient's comfort was also statistically related to the gender variable (*P* = 0.009).

## Discussion

The main hypotheses of this study were confirmed: Telemedicine acceptance and satisfaction increased among professionals exposed to this modality (who participated in it's use); Most of the Professionals who carried out telemedicine stated that they had expectations about the increased use of telemedicine as part of their future practice; Professionals' perception of patients' satisfaction was related to experienced professionals. However, it was not more significant in patients attended by more satisfied professionals. There were age and gender differences in professional satisfaction when applying telemedicine.

The mental health professionals showed, in general, an excellent clinical experience with TM, even though they had little any previous experience with it. This new model was performed through multiple calls and was carried out unexpectedly with little resources or legal regulation and no adaptation or prior training. Professionals mostly managed to reconcile family and work-life despite the pandemic circumstances, and in general, they were quite satisfied with the TM they had performed. The work was done autonomously, adapting personal preferences and peculiarities to the new task and employment situation. Older age had a positive relationship over the satisfaction with the job done, and simultaneously, professionals perceived the approval and satisfaction from most of their patients. Therefore, the higher the clinical experience, the greater the satisfaction. This could be related to more increased safety and confidence in their prolonged professional activity.

Matching previous studies, TM's satisfaction and efficiency in professionals, including psychiatrists, is high (8). Some gender differences were detected, showing that females are more likely to carry out TM (19) and are more satisfied and adapted when conducting this modality. This fact has not been described previously, but it is a well-known that females are more likely to engage in different e-health behaviors than males (20).

Professionals felt that patients were satisfied, which overlaps previous studies addressing patients' perception of the use of TM (21). There is evidence of high patients satisfaction when receiving TM in other sanitary fields (22, 23).

In general, patients and providers/professionals are satisfied with telepsychiatry services, even though providers/professionals tend to express more concerns about the telepsychiatry's potentially adverse effects. The therapeutic relationship with TM is comparable to face-to-face services in terms of reliability of clinical evaluations and treatment outcomes (24), especially in the post-COVID era when the psychopathological presentation will be different (25).

This sample identifies TM as a new and widely generalized tool that will continue to grow in the future after adopting the methods, time, resources, professional stress, and some essential features like family reconciliation. This aspect became important when considering the high prevalence of anxiety and depression due to the COVID-19 pandemic (22).

There is some data on clinical interventions in severe mental disorders and TM, such as care of patients with schizophrenia (24–26) and on the benefits and costs when applying internet cognitive behavioral therapy (I-CBT) (27). Neither of our results is comparable to previous research describing phone calls for the training of functional skills (28); the use of mobile devices to decrease hospitalizations (29) to promote treatment adherence in outpatients in remission (25). These experiences can respond to link scientific evidence and clinical reality (28). Other pathologies have received care attention/treatment through TM, including patients with obsessive-compulsive disorder (29), mental health care for patients with intercultural difficulties (30), early detection and diagnosis in patients with Alzheimer's disease or mild cognitive impairment (31), and crisis interventions (32).

The use of telepsychiatry in the COVID-19 pandemic has been scarcely discussed (3, 5, 33, 34) with some experiences among psychologist, psychotherapists and clinicians. Professionals tend to prefer face-to-face care to telematics, although their experiences with telematic care during the COVID-19 pandemic were better than expected (35, 36).

This research helps the populations living far from the mental health clinics. We can agree with some of TM's advantages, such as better quality and access to care and related cost decreased. Moreover, according to the pharmaceutical companies' plans, an increase in TM's annual growth rate (between 13 and 27%, with valuation growing over 20 billion US dollars in the next several years) is expected (3). In this sense, combining TM with psychoeducation shows greater efficacy than the regular visits in the overall functioning of patients with bipolar disorder (37).

Nevertheless, our results emphasized that not all features are positive. TM shows some inconveniences and difficulties in the real world (38). Some participants (6.8%) perceived this new model as tiring at the end of the day. Most of the sample believed telemedicine could have side effects and suggested dehumanization risks in the assistance. Chakrabarti et al. (39) referred to perceived difficulties in different areas, such as communication or building a good relationship, and uncertainty over legal, regulatory, and ethical issues could influence somewhat negative attitudes regarding this model among physicians. A possible disadvantage could be that there were many failed phone calls (around 25%); additionally, some patients were either at work or accompanied by relatives when receiving the phone call, so their availability and intimacy could not be guaranteed.

### Limitations

The use of the telephone restricts the observation of the patient's non-verbal and qualitative behavior. The gold standard of e-health services should be video-conference, but in the context of the pandemic, its use was marginal due to lack of resources. The use of the telephone was considered a previous step to digital tools despite of its limitations.

The stressful state may have influenced the professional opinions of the sample during the pandemic.

In this naturalistic study, the collected data's standard practice features would not differentiate the first consultations' weight vs. follow-ups. On the other hand, the results have been obtained after a short time of performing TM (2 months), and their evolution over time remains unknown.

The previous data acquired is not underpinned by clinical trials or pilot studies. Overall, previous works support the non-inferiority of remote psychiatric care in the evaluation and treatment compared to face-to-face care (40), not regarding the age, kind of population, or mental disorder. However, specific people (children and adolescents) do need some adjustments (41). However, we describe the difficulties of applying telemedicine in the real world.

The pandemic brought about a radical change in the way of treating patients. The priority after this has been to give normality and continuity to patients who were generally willing to return to face-to-face consultations, so no more data on satisfaction with telemedicine has been collected since that date. In the future, it would be interesting to assess whether patients and professionals would be interested in giving continuity to telematic services in a context of regular assistance. As well, the mental health of the professionals should be taken into account in order to evaluate the adaptation to the new technologies use (42, 43).

## Conclusion

Telemedicine is a tool that has been identified as a complement in clinical practice and a world-class resource in pandemic situations such as COVID-19's. It can be deployed without difficulties, although these equate to procedures, time, methods, and resources. Satisfaction between professionals was high, especially those with more clinical experience and those with previous telemedicine practice. Gender and professional differences should be carefully studied. However, some disadvantages should be considered, and perhaps therapeutic relationships could become impaired. Patient satisfaction should be contrasted, and the planning and decision-making of when to use face-to-face vs. online techniques should be widely considered.

The feasibility of performing telemedicine consultations has been generalized with all types of patients, even with a shallow level of training and preparation in high-pressure situations (lockdown). This condition has been representative of many psychiatry services around the world. Therefore, training and access to resources that allow fair use of telemedicine should be promoted; thus, the barriers in patient care would be reduced (44, 45). On the other hand, professionals' perceptions must be taken into account because they are the ones who prescribe the treatments (9–11). Additionally, it should be noted that patients' and mental health providers' satisfaction is crucial for telemedicine success (46). Therefore, a comprehensive and long term evaluation for measuring professionals' satisfaction in delivering telemedicine is vital for the future (47).

## Data availability statement

The raw data supporting the conclusions of this article will be made available by the authors, without undue reservation.

## Ethics statement

Ethical review and approval was not required for the study on human participants in accordance with the local legislation and institutional requirements. Written informed consent for participation was not required for this study in accordance with the national legislation and the institutional requirements.

## Author contributions

CR, BB-V, and NC-E conceptualized, designed, and supervised the study. DR-G, BB, and LA collected the data, performed data processing, and statistical analysis. BB, DR-G, and SG-L wrote the manuscript. CR and BB-V supervised the project. AM, MG, and LA critically revised and edited the manuscript. All authors have read and agreed to the published version of the manuscript.

## Funding

This research project was supported by Castile and León's (Spain) Regional Management of Health (GRS 2075/A/2019) Scholarship for the project Clinical characterization of psychotic symptoms and their relationship with addiction severity among patients consulting for alcohol and cocaine intake and alcohol intake. This 12-month scholarship was awarded to CR (Main Researcher) and his research team. This research project was also supported by Castile and Leon's (Spain) Regional Management of Health (GRS COVID 59/A/20) Scholarship for the project Impact and approach on the mental health of patients affected by COVID, their families, and the health professionals who care for them. This scholarship was awarded to CR (principal researcher) and his research team.

## Conflict of interest

The authors declare that the research was conducted in the absence of any commercial or financial relationships that could be construed as a potential conflict of interest.

## Publisher's note

All claims expressed in this article are solely those of the authors and do not necessarily represent those of their affiliated organizations, or those of the publisher, the editors and the reviewers. Any product that may be evaluated in this article, or claim that may be made by its manufacturer, is not guaranteed or endorsed by the publisher.
